# Demographic and Clinical Characteristics Predicting Missed Clinic Visits among Patients Living with HIV on Antiretroviral Treatment in Kinshasa and Haut-Katanga Provinces of the Democratic Republic of Congo

**DOI:** 10.3390/healthcare12131327

**Published:** 2024-07-03

**Authors:** Osaremhen Ikhile, Gulzar H. Shah, Stacy Smallwood, Kristie C. Waterfield, Dziyana Nazaruk

**Affiliations:** Jiann-Ping Hsu College of Public Health, Georgia Southern University, Statesboro, GA 30460, USA; oi00156@georgiasouthern.edu (O.I.); gshah@georgiasouthern.edu (G.H.S.); ssmallwood@georgiasouthern.edu (S.S.); dnazaruk@georgiasouthern.edu (D.N.)

**Keywords:** HIV/AIDS, ART, PLHIV, DRC, missed visits

## Abstract

Background: Patients living with HIV (PLHIV) often face challenges that contribute to missed clinical care which can impact their health outcomes. Methods: This retrospective quantitative study comprised 5338 adults living with HIV who received antiretroviral treatment (ART) for 12 months, from July 2018 to June 2019 in Kinshasa and Haut-Katanga provinces of the Democratic Republic of Congo. Descriptive statistics were computed to show the level of missed appointments for ART. Multivariable associations of clinical and sociodemographic factors with a tendency to miss scheduled visits after adjusting for the covariates were examined using multivariable logistic regression analysis. Results: Six percent of PLHIV experienced at least one missed visit while 94% did not miss any visits. A small proportion (20%) of PLHIV had a viral load ≥1000 copies/mL. PLHIV receiving ART from urban clinics showed significantly higher odds of missed visits compared to those from rural areas (AOR = 4.18, 95% CI [1.84–9.511]; *p* < 0.0001). Similarly, patients from semi-rural/semi-urban clinics showed significantly higher odds of missed visits compared to those from rural areas. (AOR = 2.57, 95% CI [1.08–6.141]; *p* = 0.03). Additionally, older PLHIV (18–34 years old) demonstrated increased odds of missed visits (AOR= 1.71, 95% CI [1.0078–2.697]; *p* = 0.02) compared to those under 18 years old. Conclusions: The findings from this study strongly suggest that there is a significant association between certain demographic factors, such as age and rurality-urbanicity, and missed visits. The study findings have implications for policy and interventions targeting PLHIV at higher risk of missed visits.

## 1. Introduction

Successful engagement in care provides opportunities for HIV screening, prophylaxis, treatment of comorbid conditions, and monitoring of antiretroviral treatment (ART) and viral load [1,2]. Uninterrupted care after HIV diagnosis offers maximum benefit from ART and increases the likelihood of viral load (VL) suppression [3,4]. Assuring continuity in care is central to public health response to the HIV epidemic which can be used to determine the success of ART programs. Lack of continuity in the treatment schedule may indicate a significant failure in the quality of care, manifesting as inefficient utilization of clinic resources [5]. Studies in the U.S. and elsewhere show that approximately 50% of newly diagnosed individuals are linked to HIV care and retained in care [6,7,8,9].

Rates of missed appointments vary across cultures. For instance, A recent report by the Centers for Disease Control and Prevention (CDC) estimates that 54% of people living with HIV (PLHIV) in the U.S. are retained in care [10]. Other studies have found lower rates of care retention among PLHIV in the U.S., citing one of the main reasonings being no-show or missed visit rates ranging from 17% to 35% [5,11,12,13,14]. Studies in other countries have revealed contrasts between resource-rich countries, such as Korea with 12.5% missed visits for PLHIV [15], and resource-limited countries such as Malawi with missed visit rates ranging from 19% to 22% [16].

The negative impact of the missed appointments for HIV care is well-documented. There is evidence that missed medical appointments are associated with poorer health outcomes [17,18]. Studies across the globe show that missed appointments, an indication of interruption in care, are associated with increased risk of mortality [2,5,9,17,19,20,21,22,23,24,25,26]. The increased risk of mortality due to inconsistent access to ART affects PLHIV in both resource-rich and resource-limited settings [4,20,27].

The number of medical appointments missed is an informative indicator of a lack of retention in care indicating interruption in care [13,14,17], and in turn, an elevated risk for poor health outcomes. Studies have shown that when PLHIV demonstrates an early suboptimal adherence to treatment schedules, HIV programs may experience challenges with these patients and their adherence to future appointment schedules [12,13,18,20,21,28]. For example, research shows that PLHIV who do not attend a recommended number of medical appointments in a year are at heightened risk of being lost to follow-up [29]. Having missed two or more visits in a year was associated with lower odds of being on ART and achieving viral suppression [30]. In a study, PLHIV who missed three or more scheduled visits had nearly four times the mortality risk than PLHIV who did not miss any scheduled visits [19]. A high number of missed visits is associated with poor ART adherence, virologic failure, and increased mortality [7,9,19,23,27].

Missed visits and non-adherence to the treatment for PLHIV have been operationalized using diverse metrics. For instance, Kay and colleagues defined a missed visit as one that was not canceled by the patient or provider at any point before the scheduled visit [21]. Following the Malawi Guidelines for Clinical Management of HIV in Children and Adults, Chirambo and colleagues designated a patient as “defaulter” when they did not report to the clinic for two consecutive months and were projected to have run out of ART medication with no information on transfer or death. On the other hand, a compliant client on ART was the one who was alive and had continued their ART treatment as scheduled [16]. Similarly, in Uganda and Zimbabwe, three measures of nonoptimal adherence included missing a dose, not responding to the adherence questionnaire, and missing a visit at least once during a preceding 6-month period, which independently predicted increased immediate mortality risk in patients only monitored using clinical symptoms [26]. A missed visit was defined as any scheduled clinic visit with an HIV medical provider that was not kept [9]. In another study, unkept appointments that were not canceled by the patient or clinic staff were labeled as missed visits and these were captured continuously (summed for the total number of missed visits) and dichotomously (0 = kept the visit, 1 = missed the visit) with more missed visits indicating worse retention in care [31]. Yet, in another study, a missed visit was defined as having no assessments within six months [4] while visit constancy was defined as visit attendance during regularly spaced intervals [19,25]. The variability in defining missed visits has implications for comparability of research findings in the context of PLHIV.

Factors associated with missed healthcare visits among PLHIV were studied using a socioecological perspective, elucidating PLHIV’s vulnerability at different levels, which included individual, interpersonal, community, system, and policy levels [32]. In this study, the individual-level factors highlighted included PLHIV’s age, gender, knowledge, attitudes, and beliefs as well as mental and physical health [32]. The interpersonal level risk factors for missed appointments included social norms stigmatizing HIV and its care, and access to diverse and supportive networks/influences [32]. The community-level determinants included geographic location, community norms, values, attitudes, & power structures [32]. The policy level determinants included government regulations and other regulatory processes about prevention strategies, service eligibility, program enrollment and service administration procedures, and laws to protect the public’s health [32].

The evidence highlighting specific demographic and clinical factors influencing the continuity of HIV care underscores the impact of age, gender, physical health, and location. In the case of youth, missed visits have been associated with decreased adherence to ART [14]. Similarly, multiple studies indicate that youth are more prone to experiencing interruptions in HIV/AIDS care [2,4,27]. Several studies have reported that males exhibit a higher likelihood of encountering some form of discontinuity in care [2,22,27], while other research suggests that being female is associated with missed visits [4,17,27]. Additionally, studies have also demonstrated that a lower CD4 cell count is a contributing factor to missed visits [4,20]. Positive health changes, particularly achieving viral suppression, are linked to a more favorable perception of one’s health, encouraging consistent treatment adherence [16]. Conversely, side effects from the initiation of ART, such as vomiting, skin rashes, and jaundice, contribute to treatment default [16]. Notably, a recent study has indicated that clinics in rural areas have lower odds of treatment retention compared to urban clinics [33].

Studies of missed visits and treatment interruption for HIV/AIDS clinics in Kinshasa and Haut-Katanga Provinces as well as in the Democratic Republic of Congo (DRC) as a whole, are very limited. The primary objective of this study is to determine the level of missed clinical care visits for HIV/AIDS among PLHIV and to examine demographics and clinical characteristics associated with missed visits in the Kinshasa and Haut-Katanga provinces of the DRC. To that end, we pursue three research questions: (1) Does the risk of missed clinical care visits for HIV/AIDS among PLHIV vary by demographic characteristics? (2) Does the risk of missed clinical care visits for HIV/AIDS among PLHIV vary by the rurality-urbanicity status of the treatment centers? and (3) Are clinical characteristics of PLHIV associated with the risk of missed clinical care visits for HIV/AIDS? An understanding of the issues prompting missed visits to the DRC can be used to effectively guide reengagement interventions.

## 2. Materials and Methods

### 2.1. Data

This is a quantitative retrospective cohort study, a type of observational study in which individuals are observed and certain outcomes are measured. This study used secondary data extracted from a routinely collected program database covering 241 HIV/AIDS clinics in Haut-Katanga and Kinshasa provinces of DRC, supported by the Centers for Disease Control and Prevention (CDC) through the President’s Emergency Plan for AIDS Relief (PEPFAR). The study data covered 23 health zones: 11 health zones in Haut-Katanga, namely, Kafubu, Kasenga, Kilwa, Pweto, Kashobwe, Kipushi, Katuba, Kisanga, Kowe, Mumbunda and Tshamilemba health zones, and 12 health zones in Kinshasa—Binza Ozone, Kimbanseke, Kingabwa, Mont Ngafula 1, Masina I, Kinshasa, Limete, Lingwala, Mate-te, Ngaba, Nsele, and Ndjili.

The study data comprised 5338 adults living with HIV who received HIV primary care services and were receiving ART from these clinics for 12 months, from July 2018 to June 2019. The choice of these secondary data was guided by reasons such as this data could be trusted, it contained the variables that addressed the issue/topic, was not outdated and the collection process was efficient and standardized. An exemption from review by the Institutional Review Board was obtained as the data is already de-identified.

### 2.2. Dependent Variable

The dependent variable was missed clinical care visits for HIV/AIDS among PLHIV (aka missed visits) for HIV/AIDS. In this study, the dichotomous variable missed visits were defined as one or more scheduled appointments within 12 months that were not kept, coded as No (0), representing no missed visits in 12 months, and Yes (1), representing one or more missed visits.

### 2.3. Independent Variables

The independent variables were Age at enrollment, Patient Sex, Province, Rurality/Urbanicity, and Viral load. Patient sex, with the categories, “male” and “female”, and age at the time of the start of ART (<18 years old, >18 years old) were demographic variables. The province variable is based on the health facility’s geographic location (Haut-Katanga and Kinshasa). The variable rurality/urbanity consisted of three categories based on the status of the health zone in which the health facility was located: “rural”, “Semi-rural/Semi-urban”, and “urban”. Rural health zones included Kafubu, Kasenga, Kilwa, Pweto and Kashobwe. The semi-rural/semi-urban consisted of Kipushi, Mont Ngafula 1, and Nsele whereas the urban zones included, Binza Ozone, Katuba, Kimbanseke, Kingabwa, Kinshasa, Kisanga, Kowe, Limete, Lingwala, Masina I, Matete, Mumbunda, Ndjili, Ngaba, and Tshamilemba [34,35]. This independent variable was coded based on numerical values as follows: 1 = ‘Urban’, 2 = ‘Rural’, and 3 = ‘Semi-rural/Semi-urban’ as identified by the population density in cities or towns within the health zone [34]. The clinical variable Viral Load Suppression was categorized as a dichotomous variable < 1000 copies/mL (suppressed) vs. ≥1000 copies/mL (not suppressed) [34].

### 2.4. Analytical Methods

Descriptive statistics for independent and dependent variables were calculated. Descriptive statistics for continuous variables were presented as means and standard deviation (SD). Categorical variables were tabulated using frequency and percentage. Covariate selection was informed by the Gelberg-Andersen Behavioral Model for Vulnerable Populations and aligned with enabling and predisposing factors outlined by the model and the review of relevant literature identifying factors associated with missed visits [32]. Factors that predicted missed visits for HIV medical care (a dichotomous variable) were analyzed using multivariable logistic regression analysis. The results of logistic regression analyses were presented as adjusted odds ratios (AORs) and 95% confidence intervals (CIs); the statistical significance was defined as a *p* ≤ 0.05. All data management and statistical analyses were performed using SAS software version 9.4 (SAS Institute Inc., Cary, NC, USA).

## 3. Results

### Clinical-Demographic Data of Studied Population

**Figure 1:**
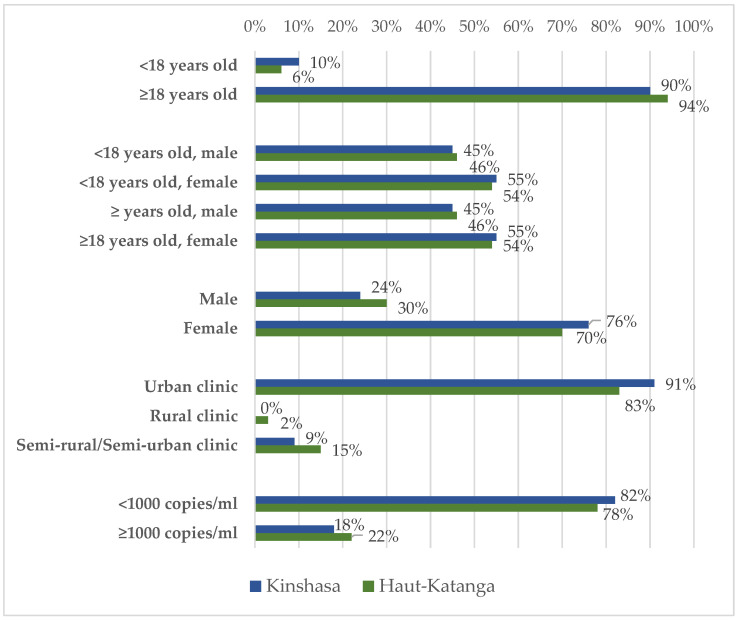
Percentage of patients within each province with at least one missed visit by Age at start of ART, gender, rurality/urbanicity, and viral load.

Six percent of patients experienced at least one missed visit while 94% experienced no missed visits. The number of missed visits (missed visit count) ranged from 0 to 11. The mean viral load for PLHIV that experienced missed visits was 22,250 copies/mL. Twenty percent of PLHIV had a viral load greater than or equal to 1000 copies/mL. Those who received treatment in clinics located in urban area clinics (76%) were a greater proportion of the study participants compared to those who received treatment in semi-rural/semi-urban (18%) and rural (6%) area clinics. Regarding age, PLHIV ≥ 18 years (89%) were most of the study participants while females < 18 years were a greater proportion compared to males < 18 years (43%) and females > 18 years were a greater proportion than males > 18 years (28%), as shown in Table 1. Differences in clinical-demographic characteristics between Kinshasa and Haut-Katanga are shown in Figure 1. 

The multivariable logistic regression analysis of having missed at least one visit within 12 months, shows that the variables age and rural-urbanicity were significantly associated with missed visits (Table 2). PLHIV receiving ART from urban area clinics had higher odds of missed visits compared to those receiving ART from rural area clinic (AOR = 4.18, 95% CI [1.854–9.511]) which was statistically significant (*p* < 0.0001). PLHIV from semi-rural/semi-urban clinics showed higher odds of missed visits compared to those from rural area clinics. (AOR = 2.57, 95% CI [1.08–6.141]; *p* = 0.03). Young adult PLHIV (i.e., 18–34 years old) had higher odds of missed visits (AOR= 1.71, 95% CI [1.078–2.697]; *p* = 0.02) compared to those younger than 18 years of age, indicating that being under the age of 18 years was protective against missed visits.

## 4. Discussion

This study showed an association between age at enrollment and missed visits. Younger PLHIV (age < 18 years), were less likely to experience missed visits compared to young adult PLHIV (18–34 years). One plausible explanation for this finding could be that young PLHIV may have more significant support structure via the family environment in comparison to those PLHIV that are 18 years or older [14] which could foster early HIV diagnosis and more active engagement in HIV care. Family-centered care that involves the family in the care and treatment plans of HIV-positive children can help to ensure that the patients are supported and that families are empowered to help their children stay healthy. The findings of the present study contrast a previous study that showed older age was protective against missed visits and interruption in care [5].

There was no association between biological sex and missed visits in this study. In the DRC and in general, women have more points of entry to care and are more likely to seek care. Thus, their familiarity with more service points providing care may also improve their chances of early referrals to HIV prevention and management programs. A study showed that males were more likely to experience some discontinuity in care [2], whereas other studies have shown that being female was associated with missed visits [4,17]. Programs targeting both male and female PLHIV may be beneficial. Male peer-to-peer individual or group initiatives should be made available and accessible to males in the DRC, particularly in high-risk groups such as men who have sex with men (MSM), sex workers, and people who inject drugs.

Rural-urbanicity was significantly associated with missed visits in this study. Like findings from this study, a recent study in DRC by Shah and colleagues showed that the odds of treatment retention were higher for PLHIV receiving ART at urban clinics [33]. Recent research has also shown that care-seeking patterns are driven by the severity of illness and access to resources, thus the sickest patents in rural and semi-rural/semi-urban areas will move to seek care in urban areas with better-equipped and better-staffed facilities [34,36]. In addition, rural communities are often more closely knit; hence, the social stigma often associated with HIV may motivate the sickest rural residents to seek HIV care at urban centers away from their communities. On the other hand, urban PLHIV have been shown to have more options and may move more frequently between clinics, interrupting their care. These moves can be complicated by the absence of interoperable patient information systems that can track PLHIV when they shift between rural, semi-rural/semi-urban, and urban areas treatment centers. This study offers additional insights into the impact of semi-rural/semi-urban areas in reference to missed visits among PLHIV, which previous studies examining missed visits have not considered.

Regarding the impact of geographic variables predicting missed visits, there was no significant association between the provinces (Kinshasa and Haut Katanga) where HIV care was being received and missed visits in this study. This further confirms the previously mentioned health-seeking behaviors of PLHIV in the DRC. Haut Katanga and Kinshasa are two different regions in the Democratic Republic of the Congo (DRC) that differ in various ways. Haut Katanga is a mining and agricultural province in the country’s southeastern part, known for its mineral-rich resources, particularly copper and cobalt. The capital of Haut Katanga is Lubumbashi, the mining capital of DRC, and the second largest city within the country. Kinshasa functions as one of the 26 provinces of the DRC, as well as the capital city of the DRC, located in the western part of the country. It is the largest city in the country, with a population of over 15 million people, and serves as the political and administrative center of the country. Haut Katanga and Kinshasa differ in demographics, culture, and economic activity. While Haut Katanga is known for its mining industry and diverse population and is largely rural, Kinshasa is a bustling urban center with a vibrant arts and entertainment scene.

Viral load prior to the start of ART is generally considered an important factor regarding predisposing PLHIV to missed visits. However, there was no association between viral load and missed visits in this study. Although conforming to the treatment schedule is known to improve viral load suppression, the risk of therapy interruptions increases with a longer duration of ART, particularly in resource-challenged environments associated with patient- and/or program-related challenges [34]. Failure to initiate treatment upon diagnosis could encourage treatment interruptions. For example, historically, protocols required a CD4 test before ART initiation [34]. Previous studies in the U.S. and Asia have shown that PLHIV with a lower CD4 cell count hence, a higher viral burden, were more prone to missed visits [4,20,27]. However, same-day ART diagnosis and treatment initiation strategy is currently being implemented in DRC as part of the “95-95-95” guidelines by UNAIDS removing the need for a CD4 test before ART initiation. Additionally factors other than ART initiation start can impact a patient’s treatment habits. For example, qualitative research from Malawi has shown that the experience of a positive change in one’s health, especially being virally suppressed could lead to a better perception of one’s health prompting defaulting positive habits towards treatment [16]. This suggests the need to employ diverse methodologies to understand the impact of viral load and ART initiation on missed visits.

A comprehensive support system linking and coordinating existing services is imperative in addressing missed visits in the DRC. An example is the Community ART Groups (CAG), which play a crucial role in defaulter tracing and encouraging attendance at health facility appointments. CAG members also organize themselves to meet and provide peer support without the involvement of the health providers. Peer support groups also offer a psychosocial component of care and address the stigma that PLHIV face. Building community capacities to provide support will ensure sustainability, continuity of interventions, and community development.

Another strategy shown to work in this setting is the decentralized drug distribution (DDD) model which allows ART patients to receive their medication refills in a community setting instead of at the health clinic. Multi-month dispensing (MMD) may also increase the chances of retention in care and prevent missed visits because compliant PLHIV do not need to return often to the facility for medication pick-up. Interventions that include more extended periods between ART refills (i.e., MMD), home delivery of ART refills, linkage to social service support programs after diagnosis, and “silent” transfer of care between clinics for PLHIV relocating (e.g., due to change in employment) are all possible successful strategies for increasing retention in care [34] and preventing the potential for missed visits. Shorter travel time to health facilities can lower the occurrence of missed visits. Telehealth visits in addition to DDD and MMD may also impact the frequency of missed visits due to geographic location.

Data linkage with community support system databases may also reduce the risk of missed visits. Maintaining interoperable patient information systems, and ongoing data linkage to detect PLHIV who show early suboptimal commitment to their HIV care and hence can receive assistance through the community services is a promising approach. Information sharing could enable the transfer of responsibility for the follow-up of PLHIV transferring between health centers. Capturing as much contact information as possible during the initial visit and updating patient contact information at every opportunity has also been suggested in the literature for enhancing retention [34].

It is important to build systems to assess the relative cost-effectiveness of different strategies developed to reduce missed visits in the face of shrinking resources. For example, access to the internet for telehealth services is still challenging even in developed countries. It may be a more significant challenge in rural areas in resource-poor countries such as DRC.

Six percent of PLHIV missed an appointment and thus experienced an interruption in treatment in this study which indicates more work still needs to be done. Continuous evidence-based quality control improvement is required to reduce the prevalence of missed visits in DRC. The demographic characteristics of age at enrollment and rural-urbanicity distinguished our study on missed visits in the multivariate model. The findings from this study raise the importance of patient tracking systems, across rural, semi-rural, semi-urban, and urban settings to ensure that PLHIV’s propensity to attend/miss clinic visits are captured. Visit attendance over the past year represents a promising simple approach to characterizing risks in the treatment continuum for PLHIV. Proactive interventions to address missed visits need to assess and focus on demographic barriers to care and the allocation of resources targeted to those at the highest risk of missed visits.

This research has several limitations. While several critical patient-level characteristics were controlled for in analyses, there may be unexplained, uncaptured variance related to unmeasured characteristics. Psychosocial factors or psychiatric conditions have been suggested to elevate the risk of missed visits for HIV medical care, but this study did not evaluate such factors. Although the sample included over 5000 patients from two CDC-affiliated clinics hence, using a venue-based sampling, the findings may not be generalizable to PLHIV at other clinics or in other countries/regions. The findings also need to be generalizable to community healthcare centers, rural clinics, or clinics outside of this setting [21,37].

## 5. Conclusions

There is evidence of associations of patient demographics with missed visits in HIV care that may be beneficial for improvements in HIV services in DRC. Public health programs involved in providing care to individuals with HIV/AIDS in the Democratic Republic of Congo should not only focus on decreasing the rates of missed visits for antiretroviral treatment, but also understand the differential risk of missed visits among subgroups of populations such as adults particularly those aged 18-34 years and PLHIV who resided in urban and semi-rural/semi-urban areas who are remarkably at higher risk of missed visits. The study findings have implications for policy and interventions targeting PLHIV at higher risk of missed visits to ensure retention in care, reduce HIV transmission, and ultimately improve the survival rate of PLHIV in the Democratic Republic of Congo.

## Figures and Tables

**Table 1:** Descriptive Statistics for Characteristics of Patients in HIV/AIDS Clinics of Kinshasa and Haut-Katanga Provinces, DRC, 2014–2019.

Patient Characteristics	Number	Percent (%)
**Age**		
<18 years old	520	11
≥18 years old	4818	89
**Biological sex**		
Male	1590	29
Female	3748	70
**Age and Sex**		
<18 years old, male	242	43
<18 years old, female	324	57
≥18 years old, male	1348	28
≥18 years old, female	3424	72
**Province**		
Kinshasa	1851	35
Haut-Katanga	3487	65
**Rural-Urban Clinic Status**		
Urban	4060	76
Rural	326	6
Semi-rural/Semi-urban	952	18
**Viral Load**		
<1000 copies/mL	4270	80
≥1000 copies/mL	1068	20

**Table 2:** Logistic regression of missed visits by PLHIV characteristics.

Patient Characteristic	AOR	95% CI		*p*-Value
		Lower	Upper	
**Age**				
18–34 years old	1.71	1.078	2.697	***0.02***
35–64 years old	1.496	0.960	2.331	0.07
65+ years old	1.262	0.286	5.575	0.76
0–17 years old	…	…	…	
**Province**				
Kinshasa	0.87	0.680	1.101	*0.24*
Haut-Katanga	…	…	…	
**Biological sex**				
Male	0.96	0.744	1.242	*0.76*
Female	…	…	…	
**Rural-Urban Clinic Status**				
Urban	4.18	1.84	9.511	***<0.0001***
Semi-rural/Semi-urban	2.57	1.08	6.141	***0.03***
Rural	…	…	…	
**Viral Load**				
≥1000 copies/mL	1.14	0.863	1.513	*0.35*
<1000 copies/mL	…	…	…	

Note. *p* = 0.05; *p*-values are highlighted in italics and bolded where statistically significant; Abbreviations: AOR, adjusted odds ratio; CI, confidence interval.

## Data Availability

The program implementing partners required that data be destroyed after publication. The authors do have data till the publication of the article. The authors can facilitate data access if requested with proper permission from the DRC Ministry of Health.

## References

[1] Israelski D., Gore-Felton C., Power R., Wood M.J., Koopman C. (2001). Sociodemographic characteristics associated with medical appointment adherence among HIV-seropositive patients seeking treatment in a county outpatient facility. Prev. Med..

[2] Batey D.S., Kay E.S., Westfall A.O., Zinski A., Drainoni M.-L., Gardner L.I., Giordano T., Keruly J., Rodriguez A., Wilson T.E. (2020). Are missed- and kept-visit measures capturing different aspects of retention in HIV primary care?. AIDS Care.

[3] Mugavero M.J., Westfall A.O., Zinski A., Davila J., Drainoni M.L., Gardner L.I., Keruly J.C., Malitz F., Marks G., Metsch L. (2012). Measuring retention in HIV care: The elusive gold standard. J. Acquir. Immune Defic. Syndr..

[4] Jiamsakula A., Kerr S.J., Kiertiburanakul S., Azwa I., Zhang F., Chaiwarith R., Wong W., Ly P.S., Kumarasamy N., Di-tangco R. (2018). Early suboptimal ART adherence was associated with missed clinical visits in HIV-infected patients in Asia. AIDS Care.

[5] Nijhawan A.E., Liang Y., Vysyaraju K., Muñoz J., Ketchum N., Saber J., Buchberg M., Venegas Y., Bullock D., Jain M.K. (2017). Missed initial medical visits: Predictors, timing, and implications for retention in HIV care. AIDS Patient Care STDs.

[6] Eaton E.F., Saag M.S., Mugavero M. (2014). Engagement in human immunodeficiency virus care: Linkage, retention, and antiretroviral therapy adherence. Infect. Dis. Clin. N. Am..

[7] Skarbinski J., Rosenberg E., Paz-Bailey G., Hall H.I., Rose C.E., Viall A.H., Fagan J.L., Lansky A., Mermin J.H. (2015). Human immunodeficiency virus transmission at each step of the care continuum in the United States. JAMA Intern. Med..

[8] Tedaldi E.M., Richardson J.T., Debes R., Young B., Chmiel J.S., Durham M.D., Brooks J.T., Buchacz K., The HOPS Investigators (2014). Retention in care within 1 year of initial HIV care visit in a multisite US cohort who’s in and who’s out?. J. Int. Assoc. Provid. AIDS Care.

[9] Safo S., Blank A., Cunningham C., Quinlivan E., Lincoln T., Blackstock O. (2017). Pain is Associated with Missed Clinic Visits Among HIV-Positive Women. AIDS Behav..

[10] Centers for Disease Control and Prevention (2021). Monitoring Selected National HIV Prevention and Care Objectives by Using HIV Surveillance Data—United States and 6 Dependent Areas.

[11] Bofll L., Waldrop-Valverde D., Metsch L., Pereyra M., Kolber M.A. (2011). Demographic and psychosocial factors associated with appointment attendance among HIV-positive outpatients. AIDS Care.

[12] Traeger L., O’Cleirigh C., Skeer M.R., Mayer K.H., Safren S.A. (2012). Risk factors for missed HIV primary care visits among men who have sex with men. J. Behav. Med..

[13] Pence B.W., Bengtson A.M., Boswell S., Christopoulos K.A., Crane H.M., Geng E., Keruly J.C., Mathews W.C., Mugavero M.J. (2019). Who Will Show? Predicting Missed Visits Among Patients in Routine HIV Primary Care in the United States. AIDS Behav..

[14] Tarantino N., Brown L.K., Whiteley L., Fernández M.I., Nichols S.L., Harper G. (2018). Correlates of missed clinic visits among youth living with HIV. AIDS Care.

[15] Kang C.R., Bang J.H., Cho S.-I. (2018). Factors Contributing to Missed Visits for Medical Care among Human Immunodeficiency Virus-Infected Adults in Seoul, Korea. J. Korean Med. Sci..

[16] Chirambo L., Valeta M., Banda Kamanga T.M., Nyondo-Mipando A.L. (2019). Factors influencing adherence to antiretroviral treatment among adults accessing care from private health facilities in Malawi. BMC Public Health.

[17] Kay E.S., Lacombe-Duncan A., Pinto R.M. (2019). Predicting Retention in HIV Primary Care: Is There a Missed Visits Continuum Based on Patient Characteristics?. AIDS Behav..

[18] Zinski A., Westfall A.O., Gardner L.I., Giordano T.P., Wilson T.E., Drainoni M.-L., Keruly J.C., Rodriguez A.E., Malitz F., Batey S. (2015). The contribution of missed clinic visits to disparities in HIV viral load outcomes. Am. J. Public Health.

[19] Mugavero M.J., Westfall A.O., Cole S.R., Geng E.H., Crane H.M., Kitahata M.M., Mathews W.C., Napravnik S., Eron J.J., Moore R.D. (2014). Beyond core indicators of retention in HIV care: Missed clinic visits are independently associated with all-cause mortality. Clin. Infect. Dis..

[20] Horberg M.A., Hurley L.B., Silverberg M.J., Klein D.B., Quesenberry C.P., Mugavero M.J. (2013). Missed office visits and risk of mortality among HIV-infected subjects in a large healthcare system in the United States. AIDS Patient Care STDs.

[21] Kay E.S., Westfall A.O. (2020). Ryan White HIV/AIDS Program recipients are more likely than non-recipients to be retained in care using six different retention measures. AIDS Care.

[22] Crawford T.N. (2014). Poor retention in care one-year after viral suppression: A significant predictor of viral rebound. AIDS Care.

[23] Waldrop-Valverde D., Guo Y., Ownby R.L., Rodriguez A., Jones D.L. (2014). Risk and protective factors for retention in HIV care. AIDS Behav..

[24] Gardner L.I., Giordano T.P., Marks G., Wilson T.E., Craw J.A., Drainoni M.-L., Keruly J.C., Rodriguez A.E., Malitz F., Moore R.D. (2014). Enhanced personal contact with HIV patients im-proves retention in primary care: A randomized trial in 6 US HIV clinics. Clin. Infect. Dis..

[25] Spinelli M.A., Scott H.M., Vittinghoff E., Liu A.Y., Gonzalez R., Morehead-Gee A., Gandhi M., Buchbinder S.P. (2019). Missed Visits Associated with Future Preexposure Prophylaxis (PrEP) Discontinuation Among PrEP Users in a Municipal Primary Care Health Network. Open Forum Infect. Dis..

[26] Kiwuwa-Muyingo S., Oja H., Walker A.S., Ilmonen P., Levin J., Mambule I., Reid A., Mugyenyi P., Todd J. (2013). Dynamic logistic regression model and population attributable fraction to investigate the association between adherence, missed visits and mortality: A study of HIV-infected adults surviving the first year of ART. BMC Infect. Dis..

[27] Zhang F., Zhu H., Wu Y., Dou Z., Zhang Y., Kleinman N., Bulterys M., Wu Z., Ma Y., Zhao D. (2014). HIV, hepatitis B virus, and hepatitis C virus co-infection in patients in the China national free antiretroviral treatment program, 2010–2012: A retrospective observational cohort study. Lancet Infect Dis.

[28] Spiegel P.B., Bennedsen A.R., Claass J., Bruns L., Patterson N., Yiweza D., Schilperoord M. (2019). Prevalence of HIV infection in conflict-affected and displaced people in seven sub-Saharan African countries: A systematic review. Lancet.

[29] Agwu A.L., Lee L., Fleishman J.A., Voss C., Yehia B.R., Althoff K.N., Rutstein R., Mathews W.C., Nijhawan A., Moore R.D. (2015). Aging and loss to follow-up among youth living with human immunodeficiency virus in the HIV Research Network. J. Adolesc. Health.

[30] Kahana S.Y., Jenkins R.A., Bruce D., Fernandez M.I., Hightow-Weidman L.B., Bauermeister J.A. (2016). Structural determinants of antiretroviral therapy use, HIV care attendance, and viral suppression among adolescents and young adults living with HIV. PLoS ONE.

[31] Zuniga J.A., García A.A., Lee J., Park J. (2020). Retention in care in aging adults with a dual diagnosis of HIV infection and type 2 diabetes mellitus: A longitudinal retrospective cross-sectional study. AIDS Res. Ther..

[32] Ikhile O. (2023). Demographic and Clinical Characteristics Predicting Missed Clinic Visits Among Patients Living with HIV on An-tiretroviral Treatment. Master’s Thesis.

[33] Shah G.H., Etheredge G.D., Nkuta L.M., Waterfield K.C., Ikhile O., Ditekemena J., Bernard B.N.B. (2021). Factors Associated with Retention of HIV Patients on Antiretroviral Therapy in Care: Evidence from Outpatient Clinics in Two Provinces of the Democratic Republic of the Congo (DRC). Trop. Med. Infect. Dis..

[34] Shah G.H., Maluantesa L., Etheredge G.D., Waterfield K.C., Ikhile O., Beni R., Engetele E., Mulenga A. (2021). HIV Viral Suppression among People Living with HIV on Antiretroviral Therapy in Haut-Katanga and Kinshasa Provinces of the Democratic Republic of Congo. Healthcare.

[35] Ewetola E., Shah G.H., Maluantesa L., Etheredge G.D., Waterfield K.C., Mulenga A., Kilundu A. (2021). Disparities in HIV Clinical Stages Progression of Patients at Outpatient Clinics in Democratic Republic of Congo. Int. J. Environ. Res. Public. Health.

[36] Shah G.H., Etheredge G.D., Schwind J.S., Maluantesa L., Waterfield K.C., Mulenga A., Ikhile O., Engetele E., Ayangunna E. (2022). Firth’s Logistic Regression of Interruption in Treatment before and after the Onset of COVID-19 among People Living with HIV on ART in Two Provinces of DRC. Healthcare.

[37] Haley D.F., Lucas J., Golin C.E., Wang J., Hughes J.P., Emel L., El-Sadr W., Frew P.M., Justman J., Adimora A.A. (2014). Retention strategies and factors associated with missed visits among low-income women at increased risk of HIV acquisition in the US (HPTN 064). AIDS Patient Care STDs.

